# Changing the face of academic medicine: an equity action plan for institutions

**DOI:** 10.1017/cts.2022.408

**Published:** 2022-05-20

**Authors:** Felicity T. Enders, Elizabeth H. Golembiewski, Minerva A. Orellana, Karen N. DSouza, Mohamed A. Addani, Eleshia J. Morrison, Joanne T. Benson, Carmen J. Silvano, Laura M. Pacheco-Spann, Joyce E. Balls-Berry

**Affiliations:** 1 Department of Quantitative Health Sciences, Mayo Clinic, Rochester, MN, USA; 2 Knowledge and Evaluation Research (KER) Unit, Mayo Clinic, Rochester, MN, USA; 3 Center for Clinical and Translational Science, Mayo Clinic, Rochester, MN, USA; 4 Department of Psychiatry and Psychology, Mayo Clinic, Rochester, MN, USA; 5 Department of Quantitative Health Sciences, Mayo Clinic, Jacksonville, FL, USA; 6 Department of Neurology, Washington University, St. Louis, MO, USA

**Keywords:** diversity, equity and inclusion, workforce diversity, health equity

## Abstract

In recent years, there have been concerted efforts to better recruit, support, and retain diverse faculty, staff, and trainees in academic medicine. However, many institutions lack comprehensive and strategic plans to provide support to retain and recruit individuals from historically underrepresented groups. In this article, we itemize specific mechanisms through which institutions can support diverse individuals with the goal of improving inclusion and belonging in the workforce to better reflect the diversity of the intended patient and research participant population.

## Introduction

Research suggests that institutions have a greater ability than individuals to challenge existing systemic barriers and create lasting changes to improve diversity, equity, and inclusion (DEI) [1], and that cultural change is critical to enhance recruitment and retention of diverse individuals [2]. Evidence also shows that diversity within clinician and scientist teams is associated with better patient outcomes [3] and greater academic impact (e.g., higher impact papers) of scientific findings [4]. Importantly, to achieve equity in healthcare and health research, we cannot rely on minority-serving institutions or historically minoritized individuals to carry the full burden because systemic problems require systemic solutions [5,6]. Instead, *all* institutions must work to achieve equity. In this paper, we present ideas for a comprehensive DEI Action Plan to assist academic medical and biomedical research institutions in initiating activities related to DEI.

### Types of Diversity

**Fig. 1.**
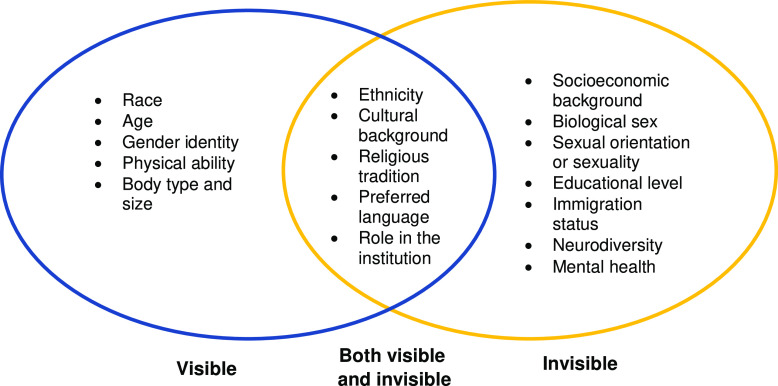
Examples of visible and invisible diversity types common in academic and medical institutions. Note: The lists above are not exhaustive, and we acknowledge that other important types of diversity may be missing. In addition, while usual visibility or invisibility is shown here, there may be variation in visibility for individual cases.

To assess DEI efforts, it is critical to specify what we mean by “diversity.” Figure 1 lists diversity types that we feel should be included. Importantly, individuals may have multiple social identities or characteristics that intersect, which may increase their vulnerability to the intensity and impact of negative experiences, leading to diminished respect and protection for and belief in these individuals.

### DEI Action Plan Items

The thirteen items below represent a comprehensive and strategic plan for change at the institutional level. These concepts were initially developed for the Center for Leading Innovation and Collaboration’s “Cohorts for Change: Building Anti-Racism Initiatives within CTSA Hubs” 2021–2022 course offering (https://clic-ctsa.org/cohorts-for-change), the goal of which is to promote structural and administrative antiracism efforts at CTSA member organizations.

### Diverse Representation across Levels

Investigate and quantify diversity among trainees and faculty, on promotion and search committees, and in leadership at the chair, director, dean, and C-suite levels. Ensure aggregate diversity data are reported in large enough categories as to avoid identifying individuals whose diversity may not be known within their work group. Avoid a single “minority” category for race and ethnicity, as members of different historically minoritized groups must contend with distinct stereotypes and prejudices [7]. Moreover, take note of the extent to which institutional diversity reflects – or does *not* reflect – the intended patient population and research participants served by the institution as well as its surrounding community.

Importantly, there is no magic diversity metric that will be the key to achieving feelings of inclusivity and belonging among diverse members of the institution. Instead, research suggests that institutions should strive to avoid the phenomenon of “only-ness” for members of diversity groups (e.g., only one woman or person of color on a committee) and continually review processes for recruiting, hiring, and promoting (see below points) [8].

### Assessment of Unconscious Bias

Nearly everyone carries unconscious bias [9], which – no matter how well-meaning the individual – can manifest in harmful microagressions or discrimination against diverse colleagues. Even when not intended, such sequelae may bear significant impact for diverse individuals, impacting the sense of belonging and over time the intent to remain in the position or institution. Awareness of one’s own unconscious bias is a necessary first step in mitigating its impact. For example, when all members of the Ohio State University College of Medicine admissions committee took the black-white Harvard Implicit Association Test (IAT), the subsequent year’s medical school class saw a 26% increase in underrepresented minority students who matriculated [10]. Findings from this and other studies indicate that awareness alone of one’s implicit bias can translate into behavior change. Therefore, all individuals at the institution should be encouraged or even required to examine their own unconscious bias using the IAT (https://implicit.harvard.edu/implicit/takeatest.html). While the IAT is not perfect, it is the only objective measure of unconscious biases across a wide range of diversity subtypes.

### Anonymous Survey

**Fig. 2.**
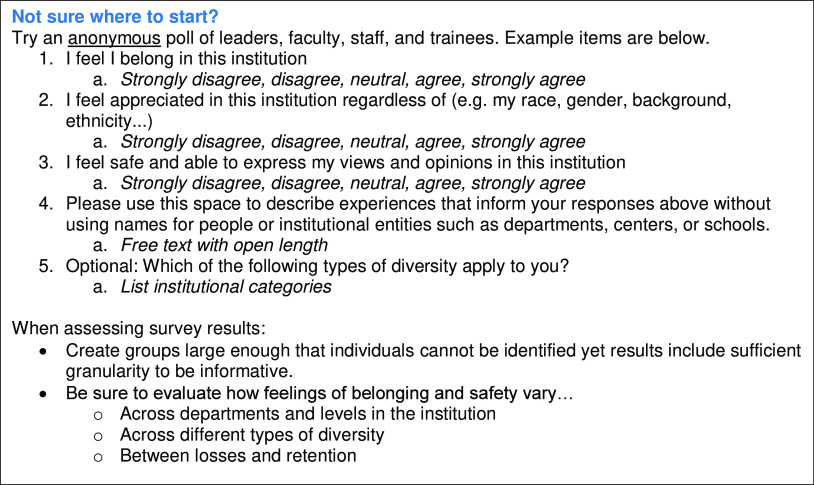
Sample survey items for anonymous polling of institutional members.

In keeping with the principles of “institutional courage” [11], which advocates for systematic, anonymous collection, and transparent reporting of abuse within institutions, anonymously survey individuals at all levels of the institution regarding their sense of belonging and inclusion (Fig. 2). Compare responses across demographics, institutional groups, and hierarchical levels. As above, avoid grouping all minority groups together. Distinguish participants by visible and invisible diversity types to further assess responses. Include open-ended options for participants to clarify their responses and provide additional feedback on opportunities to enhance DEI.

### Assess Promotion Patterns

How do promotion patterns differ by diversity? This is a critical question to assess whether issues seen at the individual level are impacting the institution. Concerns may arise at three levels:

1. Departmental leaders may have unconscious or conscious biases. For instance, women but not men may be asked to prove readiness for promotion [12]. As such, departmental “gate-keepers” may hold individuals from certain groups back from submitting a promotion packet.
2. Appointment and promotion committee members may themselves be biased. Education can help with unconscious bias from gatekeepers and committee members [13]. Committee members should also be informed on the biases that can be found in peer review letters and given examples of problematic language.
3. External peer reviewers have been shown to often incorporate bias within letters [14,15].

### Contribution to DEI in Appointments and Promotions

An individual’s contribution to DEI should be considered during appointments and promotions. Diversity statements, known more generally as contributions to DEI, are a powerful way to reward DEI efforts and partially account for the minority tax, in which diverse individuals spend more time mentoring and on committees due to the need for representation. However, contributions to DEI must be meaningfully scored and not merely symbolic [16–18]. A critical question to ask is Can an appointment or promotion advance without taking contributions to DEI into consideration? If the answer is yes, then the impact on DEI at the institution may not be as intended.

### Contribution to DEI on Annual Reviews and Performance Assessments

**Fig. 3.**
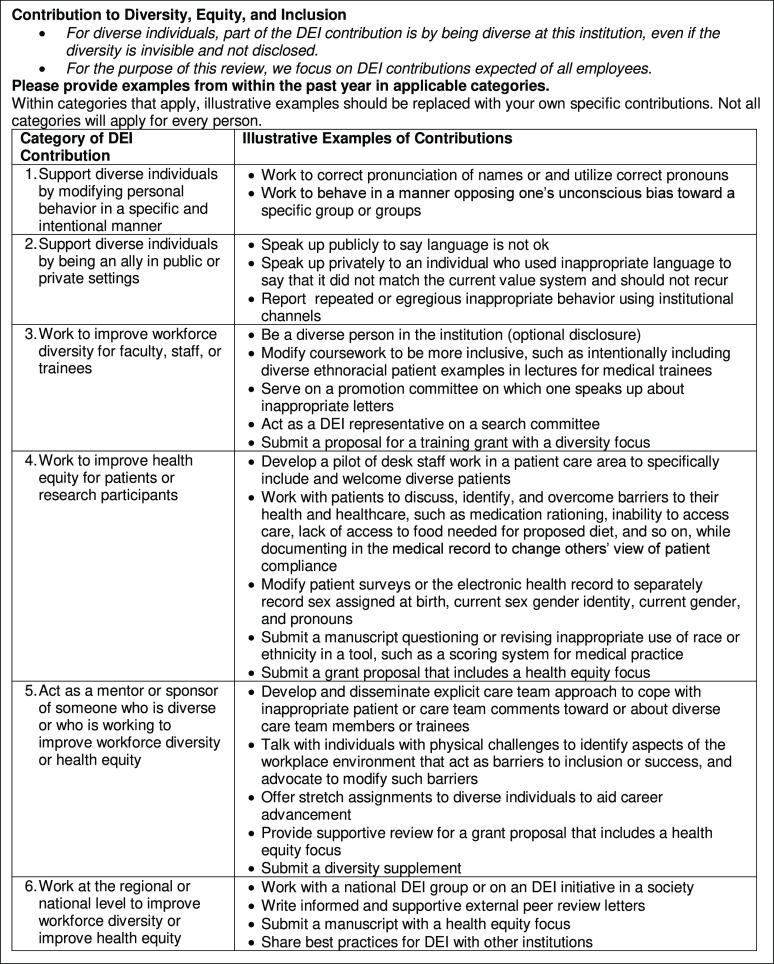
Example DEI contribution worksheet for annual review or performance appraisal.

Annual reviews should be formatted to explicitly include details on the individual’s DEI contribution. Figure 3 shows five broad areas in which individuals may have contributed to DEI; individuals should provide examples of their actions in applicable areas. Illustrative examples are included in Fig. 3.

### Funding for Research

National research funding has never achieved equity for researchers from underrepresented backgrounds [19]. Therefore, it is incumbent upon institutions to utilize national mechanisms to gain funding for diverse investigators. For example, a diversity supplement can be applied to many federal grant awards. However, the principal investigator (PI) may not know a suitable individual or know that an individual is diverse according to the NIH definition [20]. Institutions should systematically review eligible awards for inclusion of supplements and match PIs who lack a supplement with eligible candidates. Often diverse individuals are isolated within the institution, so grant writing assistance can be especially beneficial [21].

### Search Committees

Unconscious bias also impacts the recruitment and hiring process [22]. Members of search committees for trainee positions, faculty appointments, and leadership positions should be required to undergo training to minimize bias [23]. In addition, each search committee should include a person designated to speak up regarding diversity and that individual should be a voting member of the search committee. In addition, to avoid the illusion that this person can or should speak for all types of diversity based solely on their own background, there should be additional training requirements so that person is aware of issues that span multiple groups. “Fit with the institution” should be carefully assessed for bias in favor of the majority cultural groups at the institution [24].

### Retention

Gaps regarding access to research funding, mentorship, and academic promotion, and experiences of discrimination, among other factors, may contribute to attrition of diverse trainees, faculty, and staff from academic medicine [5]. Experiences of discrimination can lead to burnout [25] and adverse psychological, neural, and medical outcomes [26], suggesting that retention and professional success within this environment likely come at a high personal cost for diverse individuals[5] Strategies for retention of diverse individuals must be intentional, including, but not limited to, effective mentorship, access to networking, opportunities for professional growth (e.g., continuing education), promotion of “work/life balance,” and a welcoming, inclusive institutional culture [27]. Although a human resources representative from the organization often conducts exit interviews, departing faculty or trainees may feel reluctant to share negative experiences openly and honestly. Alternatives include an ombudsperson or a representative from the institution’s office of faculty affairs and/or office for equity and diversity. The exit interviews can serve as a critical component of a “needs assessment” to identify and elucidate organizational, structural, or cultural factors that contributed to the individual’s departure [28].

### Mentoring

All trainees, faculty, and mentors should be required to participate in mentorship training and examine their own unconscious bias. Mentorship training should include issues that are specific for diverse mentees [1]. In particular, mentors should be reminded to serve as allies for diverse trainees and provide equitable sponsorship and networking opportunities for all trainees.

### Supporting Trainees

Mentors and other faculty and staff should talk with all trainees to individually assess the impact of external barriers on their learning process. For instance, many graduate students and postdoctoral fellows face depression, anxiety, and food insecurity due to low stipends [29,30]. In particular, stipend levels are not adjusted for cost-of-living or inflation. Stipend levels aggravate diversity issues, as candidates of lower socioeconomic levels may be unable to begin or continue their graduate work [31]. Other individual issues may include: distant housing and/or lack of reliable transportation; the need for the trainee to send money home to assist their families, which is true for many trainees from international backgrounds and/or lower socioeconomic groups; difficulty prepaying for required items such as travel or other purchases and then waiting for reimbursement; and lack of appropriate network capability or device for remote access to institutional tools.

### Safe Reporting

Safe reporting is a challenge primarily because of retaliation, which may take many forms and be masked as an honest change in activity allocation. Most institutions provide avenues to report concerns anonymously. However, institutions should be aware that when an individual is the only person in their area with their diversity, traditional institutional paths may not provide protection against retaliation. Importantly, poor institutional handling of reports is a significant driver of underreporting of harassment, bullying, and other concerns in academic settings [32]. Therefore, we also recommend that institutions develop uniform processes to assess possible retaliation after the conclusion of a reported incident. Ideally, a supportive institutional official will contact the person who made the report through an in-person meeting or via phone, since efforts to collect possible retaliation in written formats are far less likely to succeed. In any follow-up, it is vital that institutional officials give greater weight to the impact of potential retaliation than to the stated intent for the action. In addition to following up in individual cases, institutions should track observed or suspected retaliation over time so that patterns of repetition can be identified and addressed.

### Institutional Standards

Comprehensively and annually review institutional standards for lack of diversity. For instance, review internal and external websites for inclusivity of photos and examples. Review application processes for excessive application fees and unnecessary standardized testing. Be transparent to all applicants about options for fee or testing waivers. Review dress codes for inclusivity of racial and ethnic individuals. Develop institutional tools for employees and patients to provide their pronouns and pronunciation of their names. Assess institutional feedback mechanisms to ensure supervisors who review feedback are trained in identifying cases in which feedback may serve as a tool for bullying a diverse person or providing feedback that is typically not given to majority individuals. Training for supervisors should include 1) when to consider excluding feedback and 2) when and how to approach an individual who provided inappropriate feedback. Train security and lobby staff to implement any security checks or procedures for medical staff in a consistent and unbiased way to avoid singling out or harassing employees who may not “look like” their idea of a medical or research professional. Establish expectations that all individuals should be treated with respect and that diverse employees and trainees should not be questioned any more than others.

### Surrounding Area

Institutions must consider the surrounding community in order to maximize recruitment and retention of diverse individuals. Are neighborhoods segregated? What practices do realtors utilize, and how do these practices differ depending on a client’s background? Are schools in the area equally strong for different neighborhoods? How equitable are schools for trainees of different diversity types? Identify these concerns and brainstorm ways that your medical institution – which likely wields enormous financial and political clout locally – can work to be a driver of inclusion and equity in the area.

## Conclusion

This list assembles concepts to help institutions identify mechanisms for enacting change and accelerating shifts in institutional culture. It is important to note that institutions should also increase diversity by hiring and admitting faculty, staff, and trainees from diverse and underrepresented backgrounds. We feel the concepts outlined in this action plan are complementary to increasing diversity targets by creating and sustaining environments that will attract diverse talent. Institutions who adopt these or similar action steps should also create an evaluation framework as a way to monitor implementation, track key outcome measures, and make process improvements where necessary. In order to carry out this work successfully, institutions need to allocate funding toward DEI efforts, including funding for leadership, staff, and the discretionary budget. In addition, DEI leadership must be integrated with institutional leadership teams to ensure bidirectional communication. We look forward to institutions bringing new and innovative tools to bear to shift the face of biomedical research to better reflect their communities, patients, and research participants.

## References

[1] Enders FT , Golembiewski EH , Orellana M , Silvano CJ , Sloan J , Balls-Berry J. The hidden curriculum in health care academia: an exploratory study for the development of an action plan for the inclusion of diverse trainees. Journal of Clinical and Translational Science 2021; 5(1): 479. 10.1017/cts.2021.867 PMC8727720 35047215

[2] Enders FT , Golembiewski EH , Pacheco-Spann LM , Allyse M , Mielke MM , Balls-Berry JE. Building a framework for inclusion in health services research: development of and pre-implementation faculty and staff attitudes toward the Diversity, Equity, and Inclusion (DEI) plan at Mayo Clinic. Journal of Clinical and Translational Science 2021; 5(1): 1. 10.1017/cts.2020.575 PMC8111694 34007470

[3] Nelson A. Unequal treatment: confronting racial and ethnic disparities in health care. Journal of the National Medical Association 2002; 94(8): 666. 12152921 PMC2594273

[4] AlShebli BK , Rahwan T , Woon WL. The preeminence of ethnic diversity in scientific collaboration. Nature Communications 2018; 9(1): 1–10. 10.1038/s41467-018-07634-8 PMC6279741 30514841

[5] Clark US , Hurd YL. Addressing racism and disparities in the biomedical sciences. Nature Human Behaviour 2020; 4(8): 774–777. 10.1038/s41562-020-0917-7 32651473

[6] Boulware LE , Corbie G , Aguilar-Gaxiola S , et al. Combating structural inequities-diversity, equity, and inclusion in clinical and translational research. The New England Journal of Medicine 2022; 386(3): 201–203. 35029847 10.1056/NEJMp2112233

[7] Siy JO , Cheryan S. Prejudice masquerading as praise: the negative echo of positive stereotypes. Personality and Social Psychology Bulletin 2016; 42(7): 941–954. 27287753 10.1177/0146167216649605

[8] Sneader K , Yee L. One is the Loneliest Number. McKinsey Quarterly [Internet], January 20, 2019. (https://www.mckinsey.com/featured-insights/gender-equality/one-is-the-loneliest-number)

[9] Morin R. Exploring racial bias among biracial and single-race adults: the IAT. Pew Research Center 2015; 19: 1–41.

[10] Capers IV Q , Clinchot D , McDougle L , Greenwald AG. Implicit racial bias in medical school admissions. Academic Medicine 2017; 92(3): 365–369. 27680316 10.1097/ACM.0000000000001388

[11] Freyd J. Official campus statistics for sexual violence mislead. Aljazeera America [Internet], 2014. (http://america.aljazeera.com/opinions/2014/7/college-campus-sexualassaultsafetydatawhitehousegender.html)

[12] Williams JC , Dempsey R. What Works for Women at Work. New York: New York University Press, 2014.

[13] Carnes M , Devine PG , Isaac C , et al. Promoting institutional change through bias literacy. Journal of Diversity in Higher Education 2012; 5(2): 63–77. 22822416 10.1037/a0028128 PMC3399596

[14] Iezzoni LI. Explicit disability bias in peer review. Medical Care 2018; 56(4): 277–278. 29432259 10.1097/MLR.0000000000000889

[15] Hengel E. Publishing while female: Are women held to higher standards? Evidence from peer review. Economic Journal 2022. DOI 10.1093/ej/ueac032.

[16] University of California. UC systemwide diversity efforts. [Internet] 2020. (https://www.ucop.edu/faculty-diversity/systemwide-efforts/index.html)

[17] Berkeley Office for Faculty Equity & Welfare. Rubric for assessing candidate contributions to diversity, equity, inclusion, and belonging. [Internet] 2022 [cited February 2022]. (https://ofew.berkeley.edu/recruitment/contributions-diversity/rubric-assessing-candidate-contributions-diversity-equity)

[18] University of Rochester School of Medicine. Diversity, equity, and inclusion. [Internet] [cited February 2022]. (https://www.urmc.rochester.edu/smd/academic-affairs/diversity-equity-inclusion.aspx)

[19] Taffe M , Gilpin N. Racial inequity in grant funding from the US National Institutes of Health. eLife 2021; 10: 7778. 10.7554/eLife.65697 PMC7840175 33459595

[20] NOT-OD-20-031 Notice of NIH’s Interest in Diversity. (NIH) NIoH; November 22, 2019, 22.

[21] Arnold L. One is a lonely demographic: minority faculty navigate institutional isolation, In. Perspectives on History: The newsmagazine of the American Historical Association, March 26, 2019.

[22] Corrice A. Unconscious bias in faculty and leadership recruitment: a literature review. AAMC Analysis in Brief 2009; 9(2): 1–2.

[23] Isaac C , Lee B , Carnes M. Interventions that affect gender bias in hiring: a systematic review. Academic Medicine 2009; 84(10): 1440–1446. 19881440 10.1097/ACM.0b013e3181b6ba00 PMC4554714

[24] Gray A. The bias of “professionalism” standards, Stanford Social Innovation Review 2019. DOI 10.48558/TDWC-4756.

[25] Hu Y-Y , Ellis RJ , Hewitt DB , et al. Discrimination, abuse, harassment, and burnout in surgical residency training. New England Journal of Medicine 2019; 381(18): 1741–1752. 31657887 10.1056/NEJMsa1903759 PMC6907686

[26] Williams DR , Lawrence JA , Davis BA , Vu C. Understanding how discrimination can affect health. Health Services Research 2019; 54(S2): 1374–1388. 31663121 10.1111/1475-6773.13222 PMC6864381

[27] Musser LR. Effective retention strategies for diverse employees. Journal of Library Administration 2001; 33(1-2): 63–72.

[28] Sengupta M , Sengupta N , Bandopadhyay K. Unravelling employee off-boarding: the magic of exit interview. International Journal of Research in Economics and Social Sciences (IJRESS) 2018; 8(1): 464–473.

[29] Bruening M , Argo K , Payne-Sturges D , Laska MN. The struggle is real: a systematic review of food insecurity on postsecondary education campuses. Journal of the Academy of Nutrition and Dietetics 2017; 117(11): 1767–1791. 28754200 10.1016/j.jand.2017.05.022 PMC6901286

[30] Forrester N. Mental health of graduate students sorely overlooked. Nature 2021; 595(7865): 135–137. 34183825 10.1038/d41586-021-01751-z

[31] Patel V. To Improve Equity, Focus on Stipends, Graduate Students Say. *The Chronicle of Higher Education* [Internet], February 17, 2014. (https://www.chronicle.com/article/to-improve-equity-focus-on-stipends-graduate-students-say/)

[32] Bergman ME , Langhout RD , Palmieri PA , Cortina LM , Fitzgerald LF. The (un) reasonableness of reporting: antecedents and consequences of reporting sexual harassment. Journal of Applied Psychology 2002; 87(2): 230–242. 12002952 10.1037/0021-9010.87.2.230

